# Tumors: Too sweet to remember?

**DOI:** 10.1186/1476-4598-6-78

**Published:** 2007-12-04

**Authors:** H Peter Vollmers, Stephanie Brändlein

**Affiliations:** 1 Institute of Pathology, University of Würzburg, Josef-Schneider-Str. 2, D-97080 Würzburg, Germany

## Abstract

Immunity, based on a natural and an educated system, is responsible for recognition and elimination of infectious particles, cellular waste, modified self and transformed cells. This dual system guarantees that dangerous particles are removed immediately after appearance and that a memory with maturated weapons exists, if the organism is re-infected by the same particle. For malignant cells, however, the immune response seems to be restricted to innate immunity, because at least for the humoral response, all so far detected tumor-specific antibodies belong to the natural immunity. In this review we try to explain why malignant cells might be "too sweet" to induce a memory.

## Immunity

Innate or natural immunity is an inherited defense system, which removes dangerous bacteria, fungi, modified self (e.g. oxidized molecules), secreted molecules, waste and transformed cells at a very early stage [1,2]. The innate response is invariable and works by using a transmitted germ-line coded pool of specific receptors [3-5]. These receptors are expressed on NK cells, γδ-T-cells and macrophages which cover a broad spectrum of different antigens [6-8]. They belong to a recently discovered family of pattern recognition receptors which show homology with the *Drosophila *Toll protein and the human interleukin-1 receptor family [3]. These Toll-like receptors (TLRs) don't recognize specific single structures, but specific patterns, termed pathogen-associated molecular patterns, like carbohydrates on glycoproteins and glycolipids and repetitive structures which are shared by different molecules and even organisms (e.g. LPS) [9,10]. This non-protein binding has another advantage, i.e. immunity does not need to follow all mutational changes, which are commonly observed for proteins. The recognition of non-self structures is, in contrast to the acquired immunity, T-cell and MHC independent.

**Figure 1:**
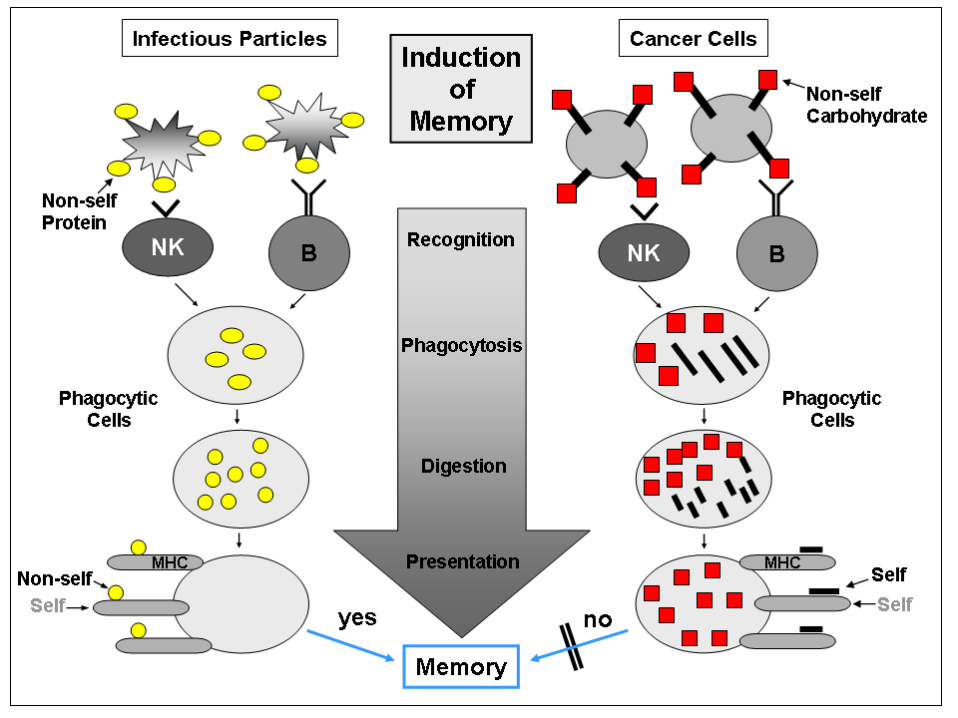
The natural immunity is the first actor on stage in immune surveillance processes. With an inherited set of pattern recognition receptors on NK cells and with natural IgM antibodies it recognizes and destroys all invasive particles and all changes and modifications within an organism. Their targets are often conservative structures, in most cases carbohydrates. Phagocytic cells clean "the battle field" and transport the garbage to nearby lymphoid organs. Here, the decision is made whether a memory should be initiated or not. In cases of infectious particles, immunocompetent Th cells (T-helper cells) are stimulated by presenting to them non-self (viral) protein peptides together with self structures (MHC). In consequence highly specific Tk (T-killer cells) and B2 cells are generated. In case of cancer cells and carbo-epitopes, this dual recognition fails, because the phagocytic cells cannot present carbohydrat structures originally seen by the innate immunity. Peptides which are associated with carbohydrate structures are "self" structures and therefore, an education and memory does normally not occur.

Based on the pioneering work of Jan Klein and Barju Benacerraf in the early 70^th^, the dual recognition system was accepted as the crucial mechanism for the induction of acquired immunity [11,12]. Non-self proteins are generally taken up by phagocytic cells, digested by proteases and presented as protein-fragments, peptides or motifs to immuno-competent cells [13]. The H-2 complex in mice and the MHC system in humans code for molecules which serve as co-receptors and which are presented together with the foreign peptide [11,14,15]. This leads to an activation of T-cells and affinity maturation of effector and memory cells and creates highly specific and high affine antibodies and receptors on T-cells. Only MHC with non-self triggers a maturation and a memory and only proteins, but not carbohydrate structures can be presented as non-self (Fig. 1).

## Cancer and sugars

In humans, malignancy can be considered as a chronic disease. Based on the simple calculation of the number of cells and the spontaneous mutation rate, cellular transformation is a common and frequent process and only the manifest tumors are rare events. A very efficient cellular repair mechanism and immuno-competent cells and mechanisms (immuno-surveillance) keep the number of growing tumors at a very low level. However, an immune response needs targets, molecules which are specifically expressed on malignant cells and absent on normal cells and tissue [5]. Over the years an enormous amount of data has accumulated clearly showing that tumor-specific epitopes associated with malignant transformation are carbohydrate modifications [16].

## Serum sugars

Sugars (oligosaccharides and polysaccharides) existing either in free form or in covalent complexes with proteins or lipids are found on all cells in an organism. These glycans are either membrane-associated and form the glycotype of a cell or are secreted. Malignant cells very often secrete specific glycans into the serum and serum measurement of certain glycan levels can be used to facilitate diagnosis, track tumor recurrence or tumor burden or provide a surrogate measure for therapeutic response. For example, the serological markers CA125, CA19-9 and CA15-3 are mucin glycoconjugates commonly over-expressed by ovarian, pancreatic and breast adenocarcinomas, respectively, and their serum levels correlate with tumor burden and prognosis [17,18]. CA19-9 is the epitope which interacts with SLe^A ^on pancreatic carcinoma mucins [19] and its expression facilitates selectin-mediated adhesion during haematogenous metastasis. In breast cancer, the tumor antigen CA15-3 is expressed on MUC1, which is aberrantly expressed in more than 90% of breast carcinomas and appears to promote invasion [20]. Therefore, the glycans CA125, CA19-9 and CA15-3 are examples of molecules that not only serve as tumor markers for diagnosis, but also appear to serve as important patho-physiological factors in cancer progression [21].

## Membrane sugars

Membrane bound carbohydrate antigens can be categorized into two major groups: (i) glycolipids such as GM2, GD2, GD3, and fucosyl GM1 (gangliosides), and Lewis^y ^(Le^y^) and globo H (neutral glycolipids); and (ii) glycoproteins such as the mucin-related epitopes Tn (GalNAcα-O-Ser/Thr), TF (Thomsen-Friedenreich, Galβ1→3GalNAcα-O-Ser/Thr) and STn (NeuAcα2→6GalNAcα-O-Ser/Thr).

Gangliosides modulate transmembrane signaling essential for tumor cell growth, invasion, and metastasis. GM2, GD2, GD3 are the major gangliosides expressed on most human cancers of neuro-ectodermal and epithelial origin [22]. In melanoma cells the ganglioside composition has been found to correlate with their metastatic potential and also to be selectively expressed in cells of a tumor mass and invading tumor cells [23]. Gangliosides have shown to be negatively correlated with survival. In melanoma naturally occurring antibodies to the ganglioside GM2 have been shown to correlate with improved survival. Lewis(y) (Le(y), also designated CD174, represents a carbohydrate blood group antigen which is strongly expressed in neoplastic gastrointestinal tissues. It has procoagulant and angiogenic activities [24].

The cell-surface glycosphingolipid Globo H is another member of a family of antigenic carbohydrates that are highly expressed on the cell surface of prostate, ovarian cancers and breast cancer cells. Furthermore, it has been established that the serum of breast cancer patients contains high levels of antibodies against the Globo H epitope [25].

The second group of membrane bound carbohydrate antigens are glycoproteins. There are several examples of specific glycoproteins undergoing changes in glycosylation upon malignant transformation, which exist in membrane-bound forms. Abnormal expression of the mucins MUC1 and MUC4 has been observed in tumor cells of various tissues, including lung, colon, pancreatic, ovarian, and breast cancers. Truncation of the *O*-glycans in cancer cells leads to the appearance of another novel carbohydrate epitopes, such as Thomas Friedrich (TF), Tn and sialyl Tn antigens [26].

Another group of membrane bound structures with modified glycosylation patterns on cancer cells are heat shock proteins. Heat shock proteins are known as chaperones which are involved in folding or maturation of proteins, transport out of the cell and signaling pathways [27,28] but they are characteristically over-expressed and modified in and on the surface of malignant transformed cells [29-31]. Over-expression of these chaperones obviously implicates a higher drug resistance and a greater degree of malignancy and is often associated with bad prognosis [32,33]. The glucose-regulated protein 78 kDa (GRP78) is a member of the heat shock protein 70 kDa (HSP70) family. High levels of cell surface-associated GRP78 are detected on a variety of carcinoma cells, such as breast cancer hepatocellular, and prostate cancers [33]. Recent studies show that GRP78 is expressed on tumor cells with a post-transcriptionally modified glycosylation [34].

Additionally, cell protection molecules and growth factor receptors are affected by tumor specific altered glycosylation.

The decay acceleration factor (DAF, also named CD55) is a cell surface molecule which prevents cell lysis by autologous complement. A modified isoform of CD55 is exclusively expressed on the membrane of stomach carcinoma cells. This modified molecule (CD55/SC-1) is co-expressed with the wild-type of CD55 on the cancer cell surface [35].

Growth factor receptors are often over-expressed on malignant cells as well. CFR-1 (cysteine-rich fibroblast growth factor receptor) is an integral membrane glycoprotein which is expressed in an post-transcriptionally modified version on almost all epithelial cancers of every type and origin and on precursor stages, but not on healthy tissues [36,37]. Again the modification is found in the carbohydrate structure of the molecule [36].

## Immunity to sugars

Antibodies and NK cells against several carbohydrate antigens have been detected in sera of cancer patients and patients treated with cancer vaccines, and have been associated with a more favorable prognosis [38]. NK cells have been detected in experimental systems binding to LewisX oligosaccharides on melanoma cells and having tumor-suppressive effects [39]. NK cells detecting the heparan sulfate moieties of membranal heparan sulfate proteoglycans (HSPGs) are able to lyse tumor cells [40].

Dendritic cells have been reported to react with colorectal cancer cells. This interaction is mediated through binding of Lewis(x) and Lewis(y) carbohydrates on CEA of colorectal cancer cells. In contrast, dendrites do not bind CEA expressed on normal colon epithelium containing low levels of Lewis antigens. This indicates that dendritic cells may recognize colorectal cancer cells through binding to tumor-specific glycosylation on CEA [41].

Furthermore, carbohydrates can be used as anti-cancer vaccines and patients develop a measurable antibody response [42,43]. Interestingly, modified carbohydrates like GD2 and GD3 lactones and N-propionylated polysialic acid were significantly more effective in inducing antibodies against tumor cells than the unmodified antigens. Tn, sTn and TF trimers (clusters) were significantly more effective than the monomers in inducing antibodies reacting with the cancer cell surface [38,42]. Patients vaccinated with tumor surface GD2 developed a significant IgM level, however, reactivity of vaccine-induced IgG antibodies against GD2 on the tumor cell surface could not be demonstrated in any patient [44].

In patients vaccinated with globo H-keyhole limpet hemocyanin vaccine a broad polyclonal antibody activity could be measured [45]. Here a significant IgG level could be detected in active sera, and the authors claim a class switch from IgM to IgG. However it could not be demonstrated that the IgG antibodies alone are responsible for the anti-vaccine activity [43].

In addition, a broad study on the humoral immunity of cancer patients revealed, that all cancer specific antibodies were germ-line coded IgMs, member of the natural immunity. Over 80% of these monoclonal antibodies were expressed by VH genes of the VH3 gene family. Within this family especially the germ-line genes DP47 and DP49 were overrepresented. Genetic restriction is not unique to heavy chain genes, it is also found within VL genes. More than 90% of the monoclonal antibodies isolated from cancer patients utilized λ-light chain genes [5,46]. Most interestingly, all investigated IgM antibodies detect carbohydrate epitopes on post-transcriptionally modified cell surface receptors [5,35,36]. The human monoclonal antibody SC-1, which was isolated from a patient with a signet ring cell carcinoma of the stomach, reacts with a N-linked carbohydrate epitope present on an isoform of DAF-B (subsequently named DAF^SC-1 ^with a molecular weight of approx. 82 kDa [35]. Clinical studies have shown that specific induction of regression and apoptosis in primary stomach cancers without any detected toxic cross-reactivity to normal tissue can be induced [47].

The human monoclonal antibody PAM-1 binds to a tumor-specific N-linked carbohydrate epitope on CFR-1/PAM-1 receptor, which is expressed on almost all epithelial cancers of every type and origin and at precursor stages, but not on healthy tissue [36,37]. PAM-1 inhibits tumor growth in vitro and in animal systems, by inducing apoptosis.

The human monoclonal antibody SAM-6 binds to a tumor-specifc isoform of grp78, a member of the hsp70 family. The epitope is an O-linked carbohydrate [34]. SAM-6 induces in malignant cells a deadly concentration of lipids, which ends in a specific form of apoptosis, namely lipoptosis [48,49].

## Conclusion

Malignant cells express and secrete tumor-specific carbohydrate structures. These modified "non-self" sugars are highly immunogenic and serve as targets for immune surveillance mechanisms. Since only peptides can be presented together with "self" to immuno-competent T-cells, no switch and education is induced and no memory is created. This makes sense, because an immunological memory is only needed for infectious particles, which can hide intracellulary and can re-infect. However, malignant cells are neither infectious nor hide, and therefore, there is no need for a memory. They really seem to "too sweet to remember" for the immune system.

## Competing interests

The author(s) declare that they have no competing interests.

## Authors' contributions

Both authors are equally contributed.

## References

[B1] Parham P (2003). Innate immunity: The unsung heroes. Nature.

[B2] Vollmers HP, Brandlein S (2005). The "early birds": natural IgM antibodies and immune surveillance. Histol Histopathol.

[B3] Medzhitov R (2001). Toll-like receptors and innate immunity. Nat Rev Immunol.

[B4] Medzhitov R, Janeway CA (2002). Decoding the patterns of self and nonself by the innate immune system. Science.

[B5] Brandlein S, Pohle T, Ruoff N, Wozniak E, Muller-Hermelink HK, Vollmers HP (2003). Natural IgM antibodies and immunosurveillance mechanisms against epithelial cancer cells in humans. Cancer Res.

[B6] Chalifour A, Jeannin P, Gauchat JF, Blaecke A, Malissard M, N'Guyen T, Thieblemont N, Delneste Y (2004). Direct bacterial protein PAMP recognition by human NK cells involves TLRs and triggers alpha-defensin production. Blood.

[B7] Akira S, Uematsu S, Takeuchi O (2006). Pathogen recognition and innate immunity. Cell.

[B8] Kaisho T, Akira S (2006). Toll-like receptor function and signaling. J Allergy Clin Immunol.

[B9] Gay NJ, Gangloff M, Weber AN (2006). Toll-like receptors as molecular switches. Nat Rev Immunol.

[B10] Pasare C, Medzhitov R (2005). Toll-like receptors: linking innate and adaptive immunity. Adv Exp Med Biol.

[B11] Klein J (1971). Private and public antigens of the mouse H-2 system. Nature.

[B12] Benacerraf B, McDevitt HO (1972). Histocompatibility-linked immune response genes. Science.

[B13] Zinkernagel RM, Doherty PC (1997). The discovery of MHC restriction. Immunol Today.

[B14] Snell GD (1952). The immunogenetics of tumor transplantation. Cancer Res.

[B15] Dausset J (1958). [Iso-leuko-antibodies.]. Acta Haematol.

[B16] Fuster MM, Esko JD (2005). The sweet and sour of cancer: glycans as novel therapeutic targets. Nat Rev Cancer.

[B17] Nakagoe T, Sawai T, Tsuji T, Jibiki MA, Nanashima A, Yamaguchi H, Yasutake T, Ayabe H, Arisawa K, Ishikawa H (2002). Difference in prognostic value between sialyl Lewis(a) and sialyl Lewis(x) antigen levels in the preoperative serum of gastric cancer patients. J Clin Gastroenterol.

[B18] Satoh H, Ishikawa H, Yamashita YT, Takahashi H, Ishikawa S, Kamma H, Ohtsuka M, Hasegawa S (1998). Predictive value of preoperative serum sialyl Lewis X-i antigen levels in non-small cell lung cancer. Anticancer Res.

[B19] Hayashi N, Nakamori S, Okami J, Nagano H, Dono K, Umeshita K, Sakon M, Narimatsu H, Monden M (2004). Association between expression levels of CA 19-9 and N-acetylglucosamine-beta;1,3-galactosyltransferase 5 gene in human pancreatic cancer tissue. Pathobiology.

[B20] Schroeder JA, Adriance MC, Thompson MC, Camenisch TD, Gendler SJ (2003). MUC1 alters beta-catenin-dependent tumor formation and promotes cellular invasion. Oncogene.

[B21] Kobata A, Amano J (2005). Altered glycosylation of proteins produced by malignant cells, and application for the diagnosis and immunotherapy of tumours. Immunol Cell Biol.

[B22] Nakamura K, Tanaka Y, Shitara K, Hanai N (2001). Construction of humanized anti-ganglioside monoclonal antibodies with potent immune effector functions. Cancer Immunol Immunother.

[B23] Fredman P, Hedberg K, Brezicka T (2003). Gangliosides as therapeutic targets for cancer. BioDrugs.

[B24] Le Pendu J, Marionneau S, Cailleau-Thomas A, Rocher J, Le Moullac-Vaidye B, Clement M (2001). ABH and Lewis histo-blood group antigens in cancer. Apmis.

[B25] Hakomori S (2002). Glycosylation defining cancer malignancy: new wine in an old bottle. Proc Natl Acad Sci U S A.

[B26] Dube DH, Bertozzi CR (2005). Glycans in cancer and inflammation--potential for therapeutics and diagnostics. Nat Rev Drug Discov.

[B27] Macario AJ, Conway de Macario E (2005). Sick chaperones, cellular stress, and disease. N Engl J Med.

[B28] Hendershot LM (2004). The ER function BiP is a master regulator of ER function. Mt Sinai J Med.

[B29] Misra UK, Gonzalez-Gronow M, Gawdi G, Pizzo SV (2005). The role of MTJ-1 in cell surface translocation of GRP78, a receptor for alpha 2-macroglobulin-dependent signaling. J Immunol.

[B30] Delpino A, Piselli P, Vismara D, Vendetti S, Colizzi V (1998). Cell surface localization of the 78 kD glucose regulated protein (GRP 78) induced by thapsigargin. Mol Membr Biol.

[B31] Ma Y, Hendershot LM (2004). The role of the unfolded protein response in tumour development: friend or foe?. Nat Rev Cancer.

[B32] Zhai L, Kita K, Wano C, Wu Y, Sugaya S, Suzuki N (2005). Decreased cell survival and DNA repair capacity after UVC irradiation in association with down-regulation of GRP78/BiP in human RSa cells. Exp Cell Res.

[B33] Li J, Lee AS (2006). Stress induction of GRP78/BiP and its role in cancer. Curr Mol Med.

[B34] Rauschert N, Brandlein  S, Holzinger  E, Hensel  F, Müller-Hermelink HK, Vollmers HP (2007). A new tumor-specific variant of GRP78 as target for antibody-based therapy.. Lab Invest.

[B35] Hensel F, Hermann R, Schubert C, Abe N, Schmidt K, Franke A, Shevchenko A, Mann M, Muller-Hermelink HK, Vollmers HP (1999). Characterization of glycosylphosphatidylinositol-linked molecule CD55/decay-accelerating factor as the receptor for antibody SC-1-induced apoptosis. Cancer Res.

[B36] Hensel F, Brandlein S, Eck M, Schmidt K, Krenn V, Kloetzer A, Bachi A, Mann M, Muller-Hermelink HK, Vollmers HP (2001). A novel proliferation-associated variant of CFR-1 defined by a human monoclonal antibody. Lab Invest.

[B37] Brandlein S, Beyer I, Eck M, Bernhardt W, Hensel F, Muller-Hermelink HK, Vollmers HP (2003). Cysteine-rich fibroblast growth factor receptor 1, a new marker for precancerous epithelial lesions defined by the human monoclonal antibody PAM-1. Cancer Res.

[B38] Slovin SF, Keding SJ, Ragupathi G (2005). Carbohydrate vaccines as immunotherapy for cancer. Immunol Cell Biol.

[B39] Chen S, Fukuda M (2006). Cell type-specific roles of carbohydrates in tumor metastasis. Methods Enzymol.

[B40] Zilka A, Landau G, Hershkovitz O, Bloushtain N, Bar-Ilan A, Benchetrit F, Fima E, van Kuppevelt TH, Gallagher JT, Elgavish S, Porgador A (2005). Characterization of the heparin/heparan sulfate binding site of the natural cytotoxicity receptor NKp46. Biochemistry.

[B41] Aarnoudse CA, Garcia Vallejo JJ, Saeland E, van Kooyk Y (2006). Recognition of tumor glycans by antigen-presenting cells. Curr Opin Immunol.

[B42] Ragupathi G, Koide F, Livingston PO, Cho YS, Endo A, Wan Q, Spassova MK, Keding SJ, Allen J, Ouerfelli O, Wilson RM, Danishefsky SJ (2006). Preparation and evaluation of unimolecular pentavalent and hexavalent antigenic constructs targeting prostate and breast cancer: a synthetic route to anticancer vaccine candidates. J Am Chem Soc.

[B43] Gilboa E (2004). The promise of cancer vaccines. Nat Rev Cancer.

[B44] Ragupathi G, Livingston PO, Hood C, Gathuru J, Krown SE, Chapman PB, Wolchok JD, Williams LJ, Oldfield RC, Hwu WJ (2003). Consistent antibody response against ganglioside GD2 induced in patients with melanoma by a GD2 lactone-keyhole limpet hemocyanin conjugate vaccine plus immunological adjuvant QS-21. Clin Cancer Res.

[B45] Wang ZG, Williams LJ, Zhang XF, Zatorski A, Kudryashov V, Ragupathi G, Spassova M, Bornmann W, Slovin SF, Scher HI, Livingston PO, Lloyd KO, Danishefsky SJ (2000). Polyclonal antibodies from patients immunized with a globo H-keyhole limpet hemocyanin vaccine: isolation, quantification, and characterization of immune responses by using totally synthetic immobilized tumor antigens. Proc Natl Acad Sci U S A.

[B46] Hensel F, Knorr C, Hermann R, Krenn V, Muller-Hermelink HK, Vollmers HP (1999). Mitogenic autoantibodies in Helicobacter pylori-associated stomach cancerogenesis. Int J Cancer.

[B47] Vollmers HP, Zimmermann U, Krenn V, Timmermann W, Illert B, Hensel F, Hermann R, Thiede A, Wilhelm M, Ruckle-Lanz H, Reindl L, Muller-Hermelink HK (1998). Adjuvant therapy for gastric adenocarcinoma with the apoptosis-inducing human monoclonal antibody SC-1: first clinical and histopathological results. Oncol Rep.

[B48] Pohle T, Brandlein S, Ruoff N, Muller-Hermelink HK, Vollmers HP (2004). Lipoptosis: tumor-specific cell death by antibody-induced intracellular lipid accumulation. Cancer Res.

[B49] Brändlein S (2007). The human IgM antibody SAM-6 induces  tumor-specific apoptosis with oxidized low-density lipoprotein. Mol Cancer Ther.

